# Spatial Heterogeneity analysis of urban forest ecosystem services in Zhengzhou City

**DOI:** 10.1371/journal.pone.0286800

**Published:** 2023-06-08

**Authors:** Yarong Yang, Jie Ma, Hong Liu, Lili Song, Wei Cao, Yifan Ren

**Affiliations:** 1 School of Horticulture and Landscape Architecture, Henan Institute of Science and Technology, Xinxiang, China; 2 Henan Province Engineering Research Center of Horticultural Plant Resource Utilization and Germplasm Enhancement, Xinxiang, China; Soil and Water Resources Institute ELGO-DIMITRA, GREECE

## Abstract

Understanding the spatial distribution of urban forest ecosystem services is essential for urban planners and managers to effectively manage cities and is an essential part of sustainable urban development. Mapping the spatial distribution of urban forest ecosystem services and improving the accuracy of its assessment scale will undoubtedly provide a more accurate reference basis for later management. In this study, we used the i-Tree Eco model and kriging interpolation to quantify and map urban forest ecosystem services and their spatial distribution in Zhengzhou, a city along the lower reaches of the Yellow River in China; analyzed the mapping errors and applicable conditions; and further explored the spatial differences using geographic probes. The i-Tree Eco model estimation results showed that the total carbon storage in the urban forest of Zhengzhou city was 75.7 tons, the annual carbon sequestration was 14.66 tons, the trees and shrubs in the urban area of Zhengzhou city could effectively avoid a total of 307.86 m^3^ of surface runoff per year, and trees and shrubs removed 411.8 kg/year of air pollution (O_3_, CO, NO_2_, PM_2.5_, PM_10_, and SO_2_). The spatial distribution of all urban forest ecosystem services showed significant heterogeneity, but the spatial evaluation precision of different factors varied. GDP and population data showed a negative correlation with ecosystem services, and ecosystem services were abundant in watershed and woodland areas. This study differs from traditional assessments based on regional data due to its improved spatial evaluation accuracy, and the results, discussion, and analysis not only help Zhengzhou’s own urban development, but also provide a basis for the future construction and management of other cities, the Central Plains urban agglomeration, and the surrounding larger regions. This will contribute to the enhancement of ecosystem services and thus improve the ecological conditions of the region. This will also have a positive effect on the health of urban residents.

## Introduction

Declining biodiversity, coupled with the degradation of ecosystem services, is a major environmental crisis facing the world today. Ecosystem services are all the benefits that humans derive from ecosystems, including provisioning services, regulating services, cultural services, and supporting services [1–3]. Green infrastructure within cities that can provide services to people is an important part of the urban ecosystem [4]. The main body of urban forest includes trees, various types of vegetation, and the natural environment; these areas are important for promoting urban ecological protection and improving the relationship between humans and nature. A growing number of studies have highlighted the contribution of ecosystem services provided by urban forests to the quality of urban life [5].

Quantifying ecosystem services provides data to support a scientific understanding of the tensions between ecosystem conservation and use. Conducting systematic research on ecosystem services from different scales of ecosystems, regions, and countries and developing methods for assessing ecosystem services are important for developing theories and methods of ecosystem service research and ensuring ecological security [6]. The application of assessment models is a major breakthrough in the field of ecosystem service quality assessment research [7–14]. Compared to traditional assessment methods, the current modeling approach is adapted to assessing ecosystems with high levels of heterogeneity and diversity such as cities. Therefore, many researchers have conducted studies on urban forests based on models [15–18]. The i-Tree model provides urban and neighborhood forestry analysis and quantitative valuation of ecological benefits [19–25]. The model is based on field research data, and it also provides a foundation for spatial heterogeneity analysis.

The spatial distribution of quantified urban forest ecosystem services has been a focus of academic discussion. The main purpose of spatial analysis is to solve geospatial problems and to obtain derived information and new knowledge from the spatial relationships between targets [26]. There are quite a few methods for the spatial analysis of ecosystem service, and different methods have their own characteristics. For example, the spatial distribution of the value of ecosystem services is derived using the zonal statistics and correlation merging method of ArcGIS [27]. Modeling methods, such as the SolVES model and the CA-Markov model, can also be used based on the Public Participation Geographic Information System (PPGIS) approach to collect survey data and spatially analyze a range of ecosystem values [28]. However, the reliability of the SolVES model’s evaluation results and transfer value prediction results and the degree of influence of natural conditions and socio-economic development factors on social values need to be comparatively analyzed [29]. In contrast, the CA-Markov model is used to simulate land use change and estimate the value of its ecosystem services [30]. However, how to integrate more efficiently and closely with ArcGIS to achieve other aspects and larger scale application development is one of the challenges the CA-Markov model will face in the near future [31]. Due to the difficulty in obtaining survey data, inaccurate data, and missing data, most studies are based on the regional scale, up to the street scale, which makes it more difficult for further more accurate spatial mapping of ecosystem services.

Geostatistics provides ecologists with an effective method for analyzing and interpreting spatial data [32, 33]. Geostatistical data are sampled from the study area and are used to analyze the variability of various natural phenomena, and this has proven to be the most effective method for studying spatial variability and spatial patterns [34]. With the development of imaging and 3S technology, ArcGIS combined with geostatistical spatial analysis is also widely used in ecosystem service mapping [35–38]. One of the difficulties of geostatistics-based spatial analysis is the accuracy of spatial data, and previous studies have encountered many obstacles due to data accuracy problems that may affect the success or failure of spatial analysis [39]. The i-Tree model provides a database that meets the scale requirements of geostatistical analysis. The second difficulty is the spatial attributes of the evaluation factors. Some researchers have applied geostatistics to the study of urban soil distribution patterns [40–43]. Our group has also made a preliminary attempt and found that the woody plant diversity [44] and carbon sink functions [45] of urban forests have significant spatial correlations and can be spatially analyzed by geostatistics. Since there are few relevant studies, and these are not yet comprehensive, further clarification is needed on how this method differs in evaluating other types of ecological services in urban forests.

In recent years, China has made great effort to develop urban forests and urban forest clusters. Zhengzhou, the capital of Henan Province, is a typical city along the lower reaches of the Yellow River and the "green core" of the Central Plains urban agglomeration. This has an obvious positive effect on the surrounding cities and shows a change in the same direction with distance [46]. Urban forest ecosystem services in Zhengzhou will not only have an impact on the city itself and the region but also on the ecological landscape pattern of the Yellow River basin and the ecological transition zones. An objective evaluation of urban ecosystem services and spatial differences under the effect of rapid urbanization is not only important for raising people’s environmental awareness and correctly handling the relationship between socio-economic development and ecological environmental protection but also helps to make forestry planning-related suggestions that will provide technological support for government departments to formulate relevant policies and implement ecological compensation [47, 48].

The objectives of this study were to (1) quantify urban forest urban ecosystem services in the study area and map their spatial distribution, (2) explore the geostatistics used to spatially analyze error differences in different urban forest ecosystem services, and (3) analyze the main drivers of spatial heterogeneity. We expect that the spatial distribution of urban forest ecosystem services in the study area is heterogeneous and may be influenced by both the degree of urbanization and the urban forest.

## Materials and methods

### Study area

Zhengzhou is the capital of Henan Province, China, located in the North China Plain (112°42’–114°14’E, 34°16’–34°58’N), and is known as the “Green City” and the “Mall.” To the north is the Yellow River; to the southeast is the Yellow and Huai Plain, and to the west is the Zhongyue Song Mountain. Zhengzhou is located in the transition zone from subtropical to temperate and has a warm temperate continental climate. The temperature is generally 31°C–38°C in summer and −10°C to 10°C in winter, with an average annual temperature of 14.4°C and an average annual rainfall of about 542 mm. Zhengzhou City belongs to the warm temperate deciduous broad-leaved forest vegetation type in terms of floral classification and is very rich in plant resources. After the survey, a total of 44 families, 73 genera, and 93 species of woody plants with a total of 2083 plants were recorded in the urban forests of Zhengzhou. These included 75 species of trees, including 22 evergreen and 53 deciduous species; 16 species of shrubs, including 12 evergreen and 4 deciduous species; and 2 species of vines, including 1 evergreen and 1 deciduous species.

The most abundant tree species in the urban forests of Zhengzhou is *Broussonetia papyrifera*, which accounts for 17.38% of the total number of trees. The second most abundant species is *Celtis sinensis*, accounting for 11.43% of the total. The third one is *Juglans regia*, which accounts for 8.59% of the total. The fourth is *Ligustrum lucidum*, accounting for 5.42% of the total. The fifth is *Cedrus deodara*, accounting for 5.33% of the total. These five species accounted for 48.15% of the total number of trees. The tree with the largest leaf area was *White poplar*. The average diameter at breast height of urban forest trees in Zhengzhou City was 13.26 cm, the average crown width was 3.95 m, and the average tree height was 5.93 m.

Zhengzhou Metropolitan Area is the core area of the Central Plains City Cluster that takes Zhengzhou as the center and promotes deep integration and inter-city linkage with Kaifeng, Xinxiang, Jiaozuo and Xuchang [49]. In 2014, Zhengzhou was awarded the title of National Forest City of China. In 2018, Zhengzhou was identified as the “green core” of China’s Central Plains Forest City Cluster. In 2019, Zhengzhou City announced the “Zhengzhou National Central City Forest Ecosystem Plan (2019–2025)” as an important special plan for the “Zhengzhou National Central City Ecological Construction Plan (2016–2025),” aiming to implement the Zhengzhou National Central City Ecological Construction Plan, build a healthy and safe forest ecosystem in the city, effectively guide the city’s ecological construction, and promote the construction of five major ecosystems (forest, wetland, watershed, farmland, and city) in an integrated manner. In the future, Zhengzhou will focus on creating a forest isolation circle around the suburbs of the city and vigorously promote tree planting and greening projects in the periphery of the main city to create a multi-species, multi-level, and multi-color urban forest landscape. According to the “Plan,” by 2025 the amount of green in the main urban area will be enhanced; the urban green coverage rate will reach more than 41%; the urban green space rate will reach more than 36%; the minimum value of green space rate in each urban area should be greater than 28.5%; the structure of plant communities will be optimized, and the green coverage of the built-up area will account for more than 75% of the trees and shrubs.

**Fig 1:**
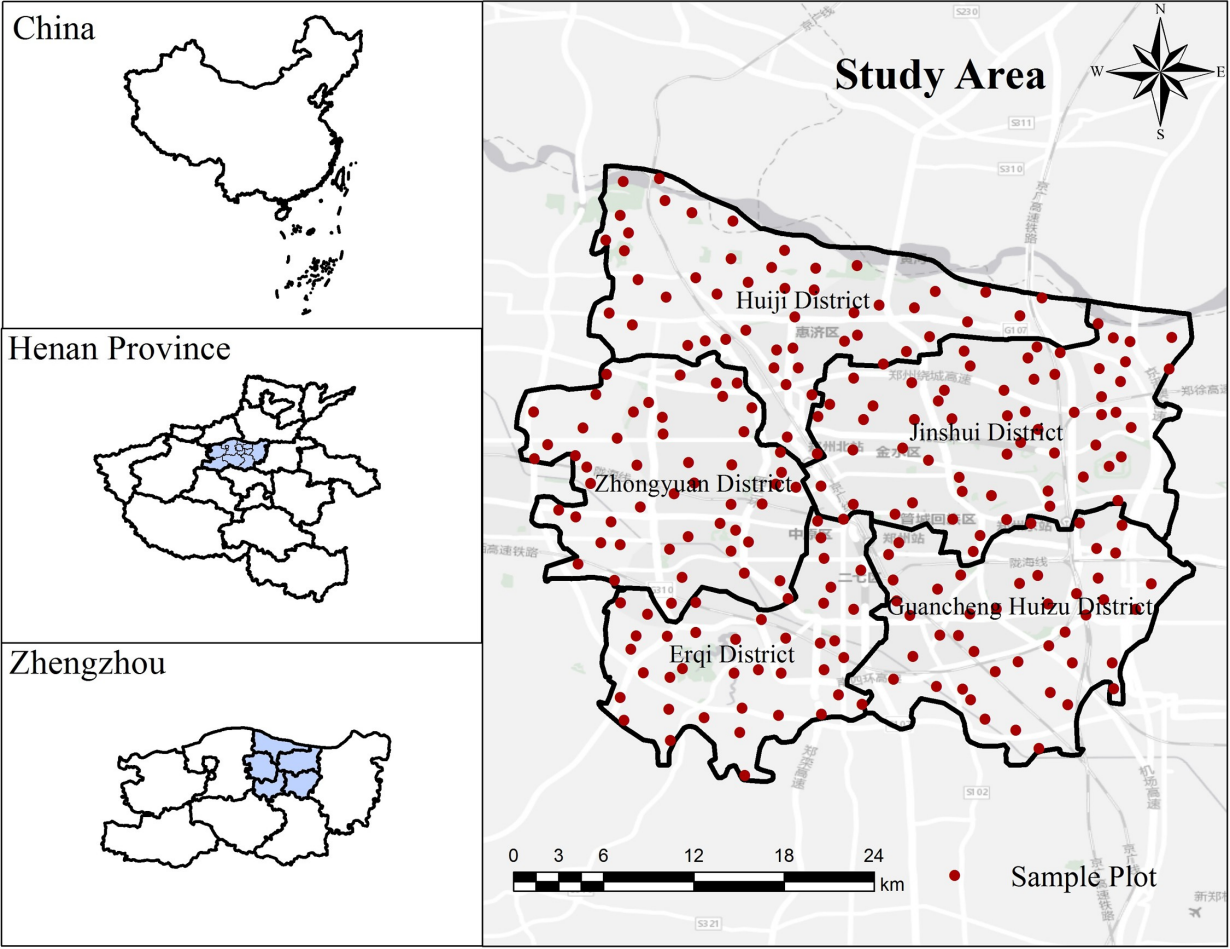
Study area map and sample point distribution (tianditu.gov.cn).

This study employed the main urban part of Zhengzhou City as the study area (Fig 1), including five administrative districts of Jinshui District, Guancheng Huizu District, Huiji District, Zhongyuan District, and Erqi District, for a total study area of about 1017 km^2^. The year-end population of the main city of Zhengzhou in 2021 was 4.635 million, with a population density of 4,557 people/ km^2^. Due to its location at the intersection of the Beijing–Guangzhou Line and the Longhai Line, Zhengzhou City is developing rapidly as a hub city that carries traffic from east to west and north to south. According to the Zhengzhou Bureau of Statistics, the built-up area of Zhengzhou within the study area from 1992 to 2019 increased from 93.1 km^2^ to 543.9 km^2^; the gross national product increased from 16.74 billion yuan to 115.8970 billion yuan, an increase of about 69.2 times, and the industrial structure was gradually dominated by tertiary industry. The urbanization rate rose from 45.2% to 74.6%, an increase of 29.4% [50].

### Establishing sample points and collecting data

In this study, the grid random sampling method was used to select the field research sample points within the study area. This method involves placing equally divided grid frame points throughout the study area, and then the sample area points for each component are randomly selected. This method balances the randomness and uniformity of the distribution.

According to the expectation of the number of sample points being greater than 200, a raster net of 2500 m × 2500 m was created, and the study area was divided into 186 grid squares. Three hundred sample points were randomly selected within the grid, and 240 sample points were finally selected after excluding inaccessible, adjacent, and similar natural sample sites (Fig 1). There were 39, 48, 43, 61, and 49 sample points distributed in Erqi, Huiji, Guancheng Huizu, Jinshui, and Zhongyuan districts, respectively. In this study, the sample sites were divided into seven categories according to land cover type as trees and shrubs, grass, buildings, roads, impervious surfaces, water bodies, and others (bare soil, farmland, vegetable fields, industrial and mining, and greenhouses) [51].

We conducted field research on the selected sample sites from June to August 2021. Each sample plot was set up as a 20 m × 20 m standard sample square, and each tree and shrub in the sample square was checked for size and photographed. The recorded data included sample plot information, vegetation information, ground cover information, and land use type. The vegetation information included species name, diameter at breast height, height, east-west crown width, north-south crown width, height under branches, missing crown rate, crown light transmission rate, building direction, distance of trees from buildings, and standing conditions.

### i-Tree Eco’s assessment of urban ecosystem services

#### Assessment items and methods

For international projects other than those in the US, Canada, Australia, Mexico, and the UK, such as this study, the localization of model parameters is required prior to using the i-Tree Eco model. This study assesses urban forest ecosystem services, including carbon storage, carbon sequestration, surface runoff avoidance, and pollution removal, in urban areas of Zhengzhou City based on localized location information, data related to annual hourly precipitation and annual hourly air quality, climate zones, species, and field measurements. Although cultural services are one of the most critical components of urban ecosystem services, i-Tree Eco currently does not include this in international projects [52]. Specific methods for assessing the above ecosystem services can be found in the UFORE method, which will be briefly described in this paper.

*Carbon storage and sequestration*. Carbon storage was estimated from biomass and carbon content, and the total tree biomass was estimated from measurements of tree diameter and height using the anisotropic equation [53, 54]. Radial growth increments, growing season length, and growth adjustment factors for canopy health and canopy light were used to estimate growth rates and thus annual carbon sequestration. The formula for calculating fresh weight biomass from carbon storage in trees was multiplied by a species- or genus-specific conversion factor to yield dry weight biomass. The difference in carbon storage estimates between year x and year x+1 is the net carbon sequestration produced each year [55].

*Avoided surface runoff*. Annual avoided surface runoff was calculated using the interception by vegetation. i-Tree Eco estimates this based on the difference between annual runoff with and without vegetation [53]. Although leaves, branches, and bark can intercept precipitation and thus mitigate surface runoff, only precipitation intercepted by leaves was considered in this analysis [56].

*Removal of pollution*. Tree cover and leaf area index were used to estimate the amount of air pollution removed. Estimated pollutants included nitrous oxide (NO_2_), ozone (O_3_), particulate matter smaller than 2.5 microns (PM_2.5_), and sulfur oxide (SO_2_). In locations more fully supported by i-Tree Eco (e.g., cities in the U.S. and Canada), tree data are merged with local pre-processed weather and air pollution concentration data to assess pollutant removal. However, in this case, since there is no default official support for Zhengzhou, we manually entered the pollution data from local monitoring stations. However, the i-Tree Eco model fails to capture the differences in particulate matter (PM) removal between species, and the removal rate of fine particulate matter (PM 2.5) is still calculated using a poorly evaluated deposition rate function. Therefore, Gaglio et al. [57] proposed an improvement to the standard model calculation by introducing a leaf trait index to differentiate the effect of species on net PM removal, and the authors also measured model results with deposited leaf PM via vacuum filtration.

#### Normalization of multiple urban forest ecosystem services

The greatest challenge in assessing multiple urban forest ecosystem services is the difficulty of combining different urban forest ecosystem service indicators based on different units (e.g., kg, t, m^3^) [58]; thus, in this study, we converted the data into dimensionless values by standardizing the data and removing unit constraints, thereby facilitating the ability to compare and weight indicators of different units or magnitudes. Normalization of data is a typical method. Normalization serves to make the features between different dimensions numerically comparable, a process that can greatly improve the accuracy of classification. The normalization used in this study was the Min-Max normalization that is calculated as:

$$X_{i}^{'}=X_{i}-X_{\min}/X_{\max}-X_{\min}.$$ (1)

The Min-Max normalization method is a linear transformation of the original data. Let MinA and MaxA be the minimum and maximum values of attribute A, respectively, and map the original value of A into a value in the interval [0,1] by the Min-Max normalization [59]. In this study, the amounts of carbon sequestration, surface runoff avoided, and pollution removed were normalized, after which the three normalized values were combined and again normalized with the aim of obtaining an integrated urban forest ecosystem service assessment.

### Spatial mapping of ecosystem services

Geostatistics can be used in ecology to analyze, recognize, and explain complex phenomena related to spatial heterogeneity, to build spatial predictive models, and to interpolate and estimate spatial data [60]. The Kriging method is a linear unbiased optimal estimation of the sample point values to be estimated based on a number of measured sample point data in a finite neighborhood of the sample point to be estimated, after careful consideration of the shape, size and spatial interposition of the sample points and the structural information provided by the variation function, and is one of the two main elements of geostatistics [61]. This method is more accurate and realistic than other traditional estimation methods, also avoids systematic errors, and yields the estimation error and accuracy; this is a major advantage of the Kriging method [62]. The spatial distribution pattern of urban forest ecosystem services was mapped using Kriging based ArcGIS 10.2 (ESRI) in the following steps [44].

#### Exploring the data

The application of the Kriging method usually requires a normal distribution of the data and spatial correlations, and the measure of spatial correlation between points in this method is the semivariance calculated as:

$$\gamma (\text{h})=\frac{1}{2\text{N}(\text{h})}\sum_{i=1}^{\text{N(h)}}{\left({\text{x}}_{\text{i}}-{\text{x}}_{\text{i}+\text{h}}\right)}^{2}.$$ (2)

Here, γ(h) is the semivariance of the distance interval; N(h) is the total number of sample pairs of the distance interval; x_i_ is the measured sample value of the location i, and x_i+h_ is the measured sample value of the first point.

The three important aspects of the semivariance function model are the nugget variance (Co), Range (a), and Sill (C). “Co” is the semivariance value at the y-intercept that indicates the non-spatially dependent variation within the data. It can be interpreted as a discontinuity in the process across space, or as an experimental error. The parameter “a” is the distance at which the data pairs remain spatially correlated; “C” is the semivariance value of the semivariogram. “Co/C,” expressed as a percentage, is used to classify the spatial correlation. A value < 25% indicates strong spatial correlation; 25%–75% indicates moderate spatial correlation, and a value > 75% indicates weak spatial correlation [63]. Regarding this method, a clearer illustration is available in a study by Li et al. [44].

#### Modeling semivariogram

The data obeying the normal distribution were simulated as a semi-covariance function, and the best semi-covariance function model for the study area was derived by iterative comparison. The values were calculated by the following formula:

$$\text{Z}\left({\text{x}}_{0}\right)=\sum_{\text{i=1}}^{\text{n}}\lambda \text{i}\;\text{Z}\left({\text{x}}_{\text{i}}\right)$$ (3)

where Z(x_0_) is the predicted value of the spatially located point x_0_; λi is the weight, and Z(x_i_) is the observed value located at x_0_. The key to interpolation is to determine the values that optimize λi. The semi-covariance function model can be used to establish the spatial relationships of the data set and determine the weights of adjacent samples. After plotting the semivariance function, we chose the spherical model to fit the semivariogram and predicted the unknown values by iterative trials [64].

#### Accuracy inspection (fitting the optimal model)

Ordinary Kriging fits the optimal theoretical model by calculating the variance function [65]. The grid spacing is usually a good indicator of the Lag step value when the sample is located on a sampling grid. However, the parameters Lag step and maximum step (maximum value of separation distance) should be set if irregular or random sampling schemes are used to obtain the data. The Lag step value is then multiplied by the number of step groups, which should be about half of the maximum step length between all points. In the analysis of the variance function, the maximum distance of the study area (about 43905 m) was taken as the maximum step value. In addition, if the range of the fitted semi-variance function model is very small relative to the range of the empirical semi-variance function, the Lag step value can be reduced. Conversely, if the range of the fitted semi-variance function model is large relative to the range of the empirical semi-variance function, the value of the Lag step can be increased. Another way to determine the Lag step value is to use the average nearest neighbor tool to determine the average distance between a point and its nearest neighbor. This provides a fairly good Lag step value, since each Lag step value has at least a few pairs of points. This study determined the step values for each urban forest ecosystem service based on the analysis of available data.

The accuracy of Kriging interpolation is mainly affected by the choice of the theoretical model of the optimal variance function, the step size, and the number and range of neighboring points. The prediction accuracy of the model can be assessed by a cross-validation process, in which each measurement is removed from the sample pool in turn and the remaining measurements are used to estimate the error between the predicted and measured values. The accuracy of the model is verified by the error values, and the optimal model should meet the following conditions [66, 67]. The 240 samples were divided into interpolation (216, 90%) and validation (24, 10%) sets. The mean error (ME) and root mean square error (RMSE) were used to evaluate the results. ME is calculated to quantify the systematic deviation between the predicted and observed dates; RMSE can reflect the valuation sensitivity and extreme value effect of the sample data. As ME approaches 0, the smaller the RMSE, the higher the prediction accuracy of the Kriging model.


$$\text{ME}=\frac{1}{\text{n}}\sum_{\text{i=1}}^{\text{n}}\left({\text{x}}_{\text{i}}-{\text{x}}_{\text{i}}^{\wedge }\right),$$ (4)

$$\text{RMSE}=\sqrt{\frac{1}{\text{n}}\sum_{\text{i=1}}^{\text{n}}\left({\text{x}}_{\text{i}}-{\text{x}}_{\text{i}}^{\wedge }\right).}$$ (5)

Here, x_i_ and ${\text{x}}_{\text{i}}^{\wedge }$ denote the measured and estimated values, respectively, and n is the number of validation sets.

#### Hot spot analysis

After mapping the spatial distribution of urban forest ecosystem services, the ArcGIS spatial “hot spot” detection method (Hotspot Analysis-Getis-Ord Gi*) was used to reveal the spatial variation of urban forest ecosystem services [68]. Analysis of spatial clustering of high or low values of ecosystem services shows that fragmented and non-concentrated patches are continuously eliminated with increasing distance thresholds, and patches that are close together and connected are clustered to form larger patches [69]. The formulae are as follows:

G(d)*=∑j=1nWij(d)Xj∑j=1nXj, (6)

$$\text{Z}(\text{Gi}*)=(\text{Gi}*-\text{E}(\text{Gi}*))/\sqrt{\mathrm{var}(\text{Gi}*)},$$ (7)

where G* is the test coefficient for general spatial agglomeration defined by the range of distances. Wij(d) is the spatial weight defined by the distance. Xi and Xj are the observed values for region i and region j, respectively. Z(Gi *) is the normalization of G(d)*.E(Gi *) and var(Gi *) are the expected value and variance of G(d) *.Z(Gi *) was used to determine whether G(d) * satisfied a particular significant value and whether there was a positive or negative spatial correlation. When G(d) * is positive and Z(Gi *) is statistically significant, the values around region i are higher, indicating that region i is a “hot spot” for high-value clustering. When G(d) * is negative and Z(Gi *) is statistically significant, the values around region i are low, indicating that region i is a “cold spot” of low-value clustering.

### Analysis of influencing factors

#### Mechanistic approach using Geodetector

Regarding the analysis of the influencing factors, this study used Geodetector software developed based on Excel for quantitative analysis [70]. This software is often used to detect the spatial anisotropy of a phenomenon and its driving mechanism [71–73]. Spatial heterogeneity is quantified by establishing statistical relationships between variables and thus identifying possible causal relationships [74], i.e., detecting the spatial heterogeneity of the dependent variable and the extent to which the independent variable X reveals the heterogeneity of the dependent variable Y. Geodetector contains four sub-detectors, a factor detector, an ecological detector, an interaction detector, and a risk detector, and the metrics, core ideas, and main objectives of each sub-detector are quite different [75]. Combined with the research objectives of this paper, we used a factor detector and an interaction detector. The factor detector measures the degree of influence of different factors on ecosystem services and is calculated as follows:

$$q=1-\sum {\;}_{m=1}^{n}N_{m}\sigma_{m}^{2}/N\sigma^{2}.$$ (8)

where q is the degree of influence of an influence factor on the spatial distribution of urban forest ecosystem services; m is the classification of the influence factor; n is the classification of the influence factor, N_m_ and N are the spatial distribution of urban forest ecosystem service in sub-region m and the whole region, and $\sigma_{\text{m}}^{2}$ and σ^2^ are the discrete variances of the spatial distribution of urban forest ecosystem services in sub-region m and the entire region. The value of q reflects the degree of influence of each factor on the spatial distribution of urban forest ecosystem services, and its value range is [0, 1]; a larger the value means a greater degree of influence and vice versa. When the value is equal to 0 or 1, this means that the factor does not influence or completely controls the spatial distribution characteristics of urban forest ecosystem services.

The interaction detector identifies the interaction effects between different factors as a way to determine the type of impact that the joint action of two factors A and B has on the spatial distribution of urban forest ecosystem services. The evaluation criteria are shown in Table 1 [71].

**Table 1:** Interaction between A and B.

Judgment basis	Interaction contribution
q(A∩B)<Min(q(A),q(B))	Nonlinear weakening
Min(q(A),q(B))<q(A∩B)<Max(q(A),q(B))	Single-factor nonlinear weakening
q(A∩B)>Max(q(A),q(B))	Two-factor enhancement
q(A∩B) = q(A),q(B)	Independence
q(A∩B)>q(A),q(B)	Nonlinear enhancement

#### Selection of driving factors

Urban forest ecosystem services are influenced by natural-social factors [76], among which natural factors include temperature, precipitation, and topography. However, since the five districts of Zhengzhou are located in the same plain, the latitude span is small, and the temperature and precipitation are basically the same, the influence of natural factors on urban forest ecosystem services was not considered. Moreover, in general the impact of natural factors on urban forest ecosystem services takes a long time to unfold and has little effect in the short term. In contrast, social factors have a rapid and dramatic impact on urban forest ecosystem services. Based on the above reasons and the accessibility of data and the meaning of each indicator, we comprehensively analyzed and selected representative indicators and finally selected six drivers: total population reflecting the demographic status, construction land area reflecting the degree of construction, primary industry GDP, secondary industry GDP and tertiary industry GDP reflecting the economic status, and the land use ratios of forest, garden, and grassland. Data on total population, construction land area, and GDP of primary, secondary, and tertiary industries were obtained from the Zhengzhou Statistical Yearbook 2018, and data on land use ratio of forest, garden, and grassland were obtained from the National Land Survey main data bulletin of each district’s government. After the data of each area were organized, the natural fracture classification method in ArcGIS was used to classify the data into five categories, and then sampling points were established within the study area. The points were assigned to different data, and finally the analysis was performed according to the sampling points.

## Results

### Urban forest ecosystem service estimation results

In this study, the land cover type occupancy test was conducted on 240 sample squares from the study area. A grid of 1000 m × 1000 m and 16305 sample points were created within the range; each sample point was visually interpreted for land cover type, and the interpretation results showed 20.4%, 7.1%, 15.1%, 11%, 8.2%, 4.1%, and 33.9% for trees and shrubs, grasses, buildings, roads, impervious surfaces, water bodies, and others, respectively. The error of 9.3% from the calibration sample points of the study area, a result that met the software application conditions [77].

The i-Tree Eco model estimates showed that the canopy cover of the study area was 22.3% and provided approximately 12.98 ha of leaf area. The average tree density was 214 trees/ha, with the highest tree density being in Huiji District, followed by Erqi District and Jinshui District. Zhengzhou’s urban forest is a mixture of native and exotic tree species, with approximately 67% of the trees being native Asian species.

**Fig 2:**
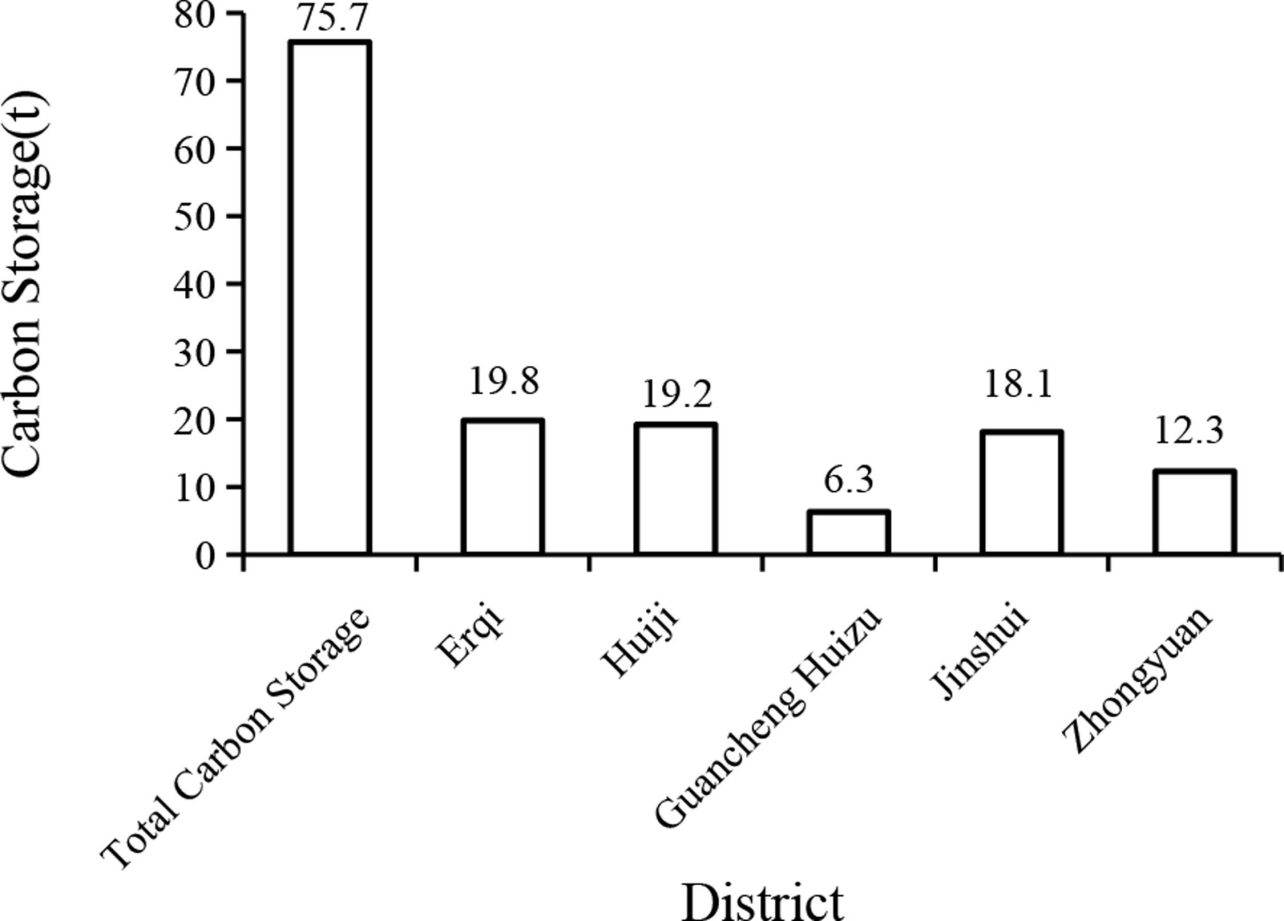
Carbon storage map of Zhengzhou urban districts.

The total carbon storage in the urban forest of Zhengzhou city was 75.7 tons (Fig 2). Erqi District had the most carbon storage, accounting for 26.2%; Guancheng Huizu District had the least carbon storage, accounting for 8.3%. The highest value in each sample with trees was 10,987.1 kg associated with agricultural land, while the lowest value in the sample with trees was 1.4 kg for unit green space. The average value of all samples with trees was 656.3 kg, and the average value of all samples was 314.68 kg.

**Fig 3:**
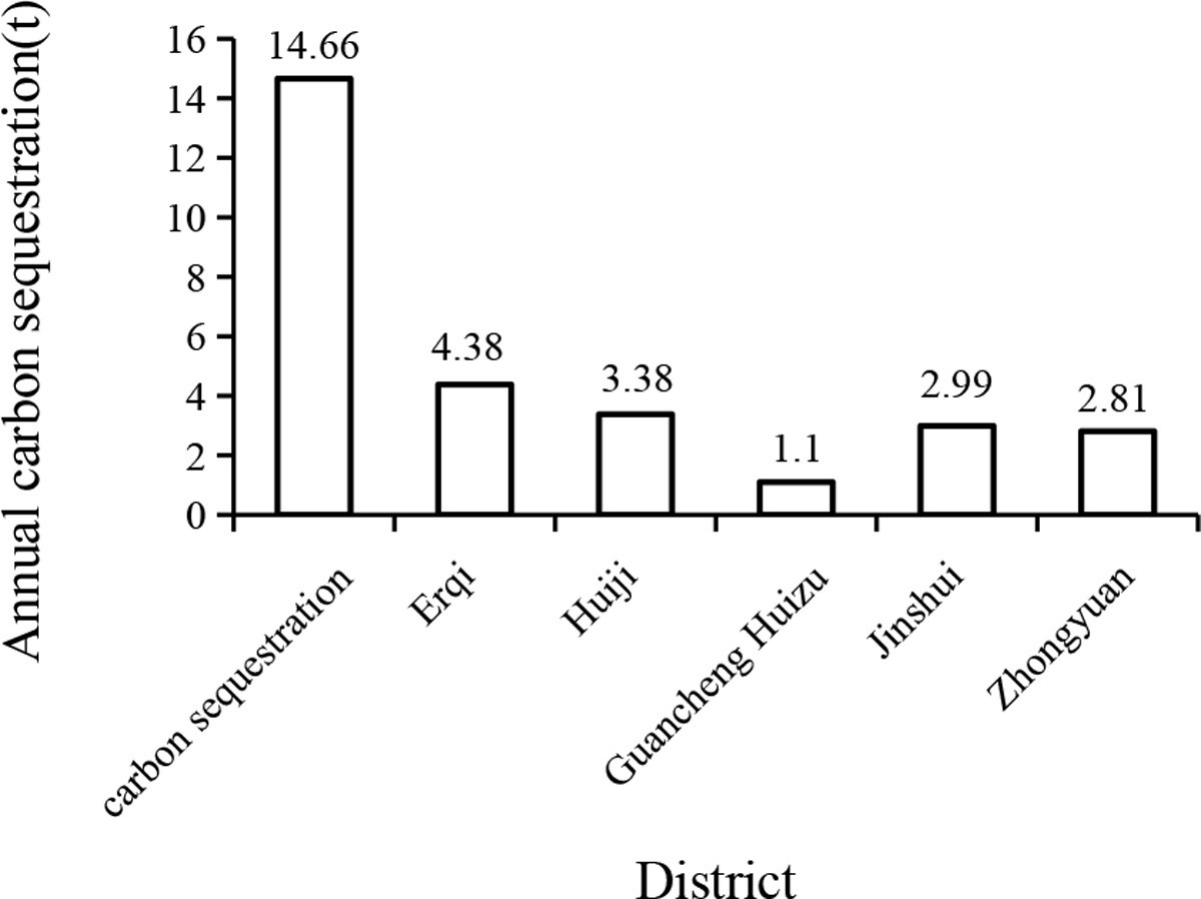
Annual carbon sequestration stock map of Zhengzhou urban districts.

The annual carbon sequestration in urban forest of Zhengzhou city was 14.66 tons (Fig 3). Erqi District contributed the most, accounting for 29.8% of the total; Guancheng Huizu District had the least annual carbon storage, accounting for 7.5%. The highest value of annual carbon sequestration was 1718.5 kg in each sample with trees in agricultural land, and the lowest value was 1.4 kg in the sample with trees associated with water surfaces. The average value of all the samples with trees was 126.1 kg, and the average value of all samples was 60.9 kg.

**Fig 4:**
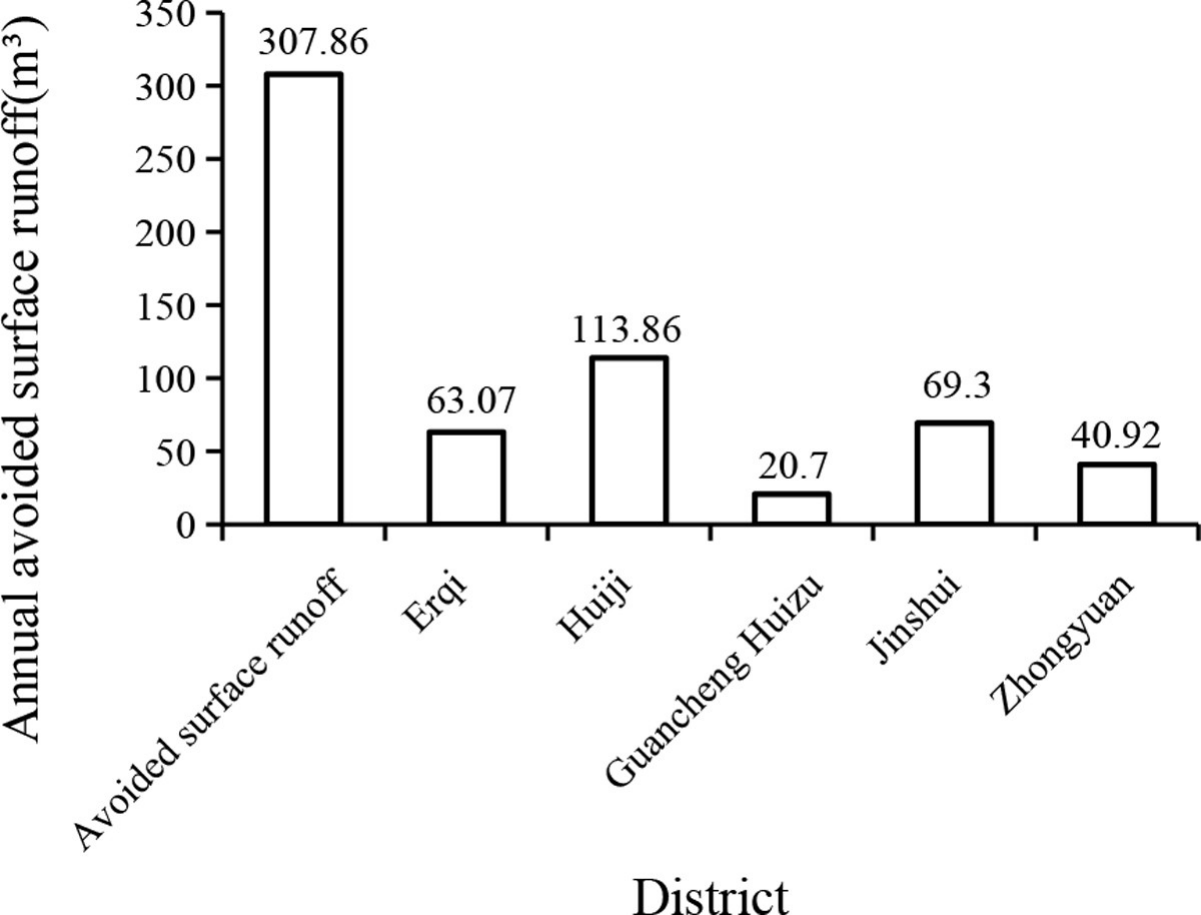
Annual avoided surface runoff map of Zhengzhou urban district.

The trees and shrubs in the urban area of Zhengzhou City can effectively avoid a total of 307.86 m^3^ of surface runoff per year (Fig 4). The annual avoided surface runoff volume in Huizu District was the highest, accounting for 36.9%; Guancheng Huizu District had the lowest annual avoided surface runoff volume, accounting for 6.7%. The highest value of annual avoided surface runoff was 34.4 m^3^for each tree sample in the study area for agricultural land, and the lowest value of was 0.1 m^3^for 10 tree samples from road green space, unit green space, and agricultural land. The average value of all samples with trees was 2.8 m^3^, and the average value of all samples was 1.2 m^3^.

**Fig 5:**
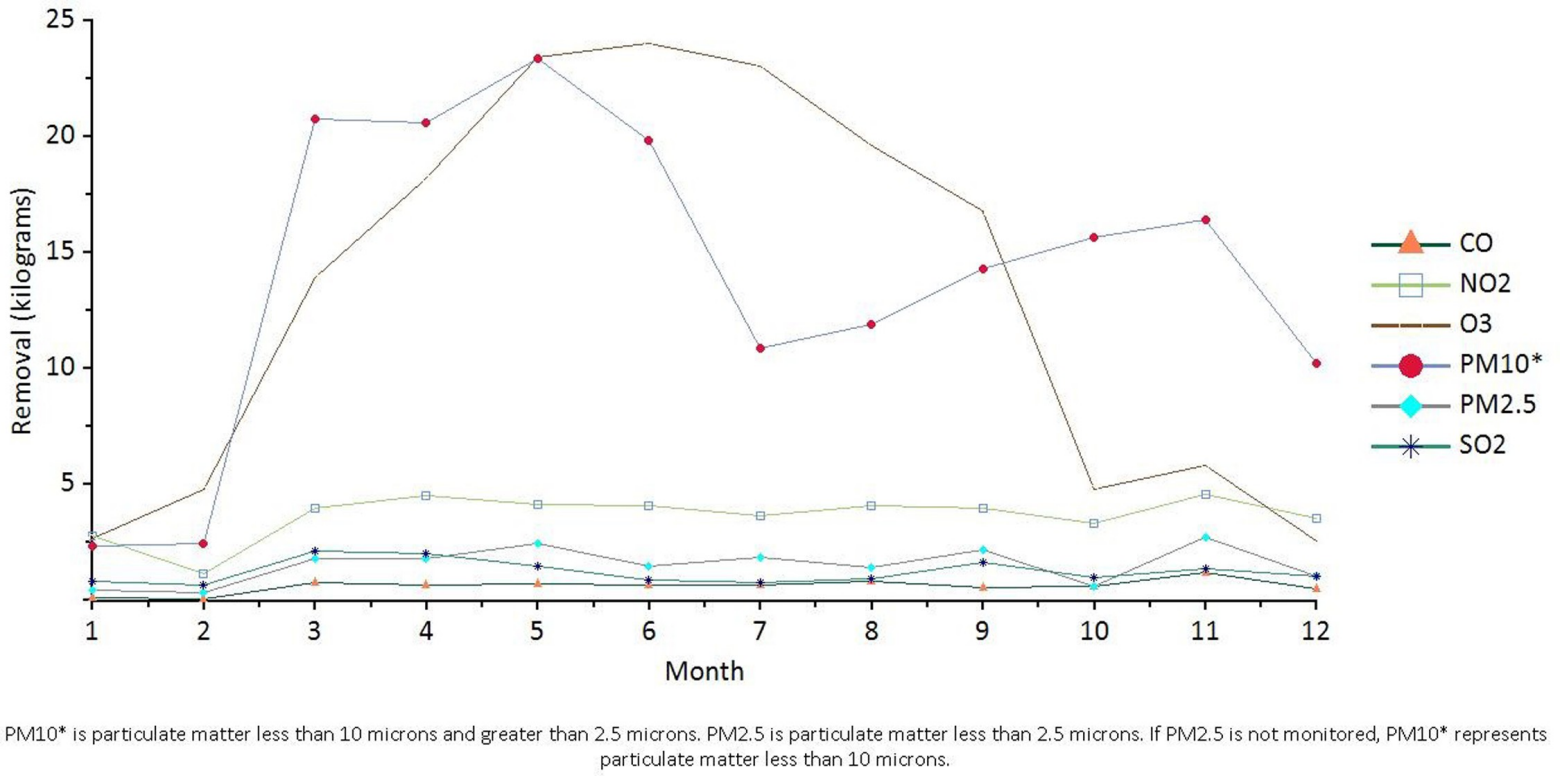
Change chart of annual pollution removal amount in Zhengzhou urban area (i-Tree Eco model out of the map).

Trees and shrubs were estimated to remove 411.8 kg/year of air pollution (O_3_, CO, NO_2_, PM2.5, PM10, and SO_2_), with the most pronounced seasonal variation in PM10 and O_3_, although both showed an overall trend of increasing and then decreasing. PM10 removed the highest amount of pollution in spring and autumn, while O_3_ removed the highest amount of pollution in summer; this may be related to the local monsoon climate (Fig 5). While removing pollution, trees in Zhengzhou’s urban forest emitted about 155.5 kg/year of volatile organic compounds (VOCs) (116.8 kg isoprene and 38.66 kg monoterpenes). Emissions varied depending on species characteristics (e.g., some genera such as oak are high isoprene emitters) and leaf biomass. Sixty-two percent of VOC emissions in urban forests were from poplar and loblolly pine trees. These VOCs are precursors to ozone formation. The highest value of annual pollution removal was 39447.5 g in each of the tree samples in the study area for agricultural land; the lowest value was 7.6 g in the tree samples for land in the area to be developed, and the average values were 3022.9 g for all tree samples and 1461.1 g for all samples.

### Spatial patterns of urban forest ecosystem services

#### Data inspection results

After exploratory analysis of the data, carbon storage, carbon sequestration, surface runoff avoidance, and pollution removal were all normally distributed after logarithmic transformation. “Co/C” for carbon storage was 21.31%, indicating a strong spatial correlation with a skewness/kurtosis of −0.69325/4.4995; “Co/C” for carbon sequestration was 21.27%, indicating a strong spatial correlation with a skewness/kurtosis of −0.10985/3.5564; “Co/C” for surface runoff avoidance was 30.48%, indicating moderate spatial correlation, with skewness/kurtosis of −0.66629/2.6439; “Co/C” for pollution removal was 60.33%, with skewness/kurtosis of −0.66629/2.6439; “Co/C” was 60.33%, indicating medium spatial correlation with a skewness/kurtosis of −0.4516/2.9347.

The average nearest neighbor tool was used to determine the Lag step value (1369 m) for each urban forest ecosystem service; this was used to determine the number of step groups as 15, and the accuracy of the model fit was also high.

The MEs for carbon storage, carbon sequestration, surface runoff avoidance, and decontamination were 0.0785, 0.1230, 0.0103, and 0.0490, respectively, and the RMSEs were 0.7499, 1.0373, 0.2223, and 0.5849, respectively. The spatial model for surface runoff avoidance had the lowest MEs and RMSEs, indicating that surface runoff avoidance yielded a higher prediction accuracy.

#### Single ecosystem service assessment results

**Fig 6:**
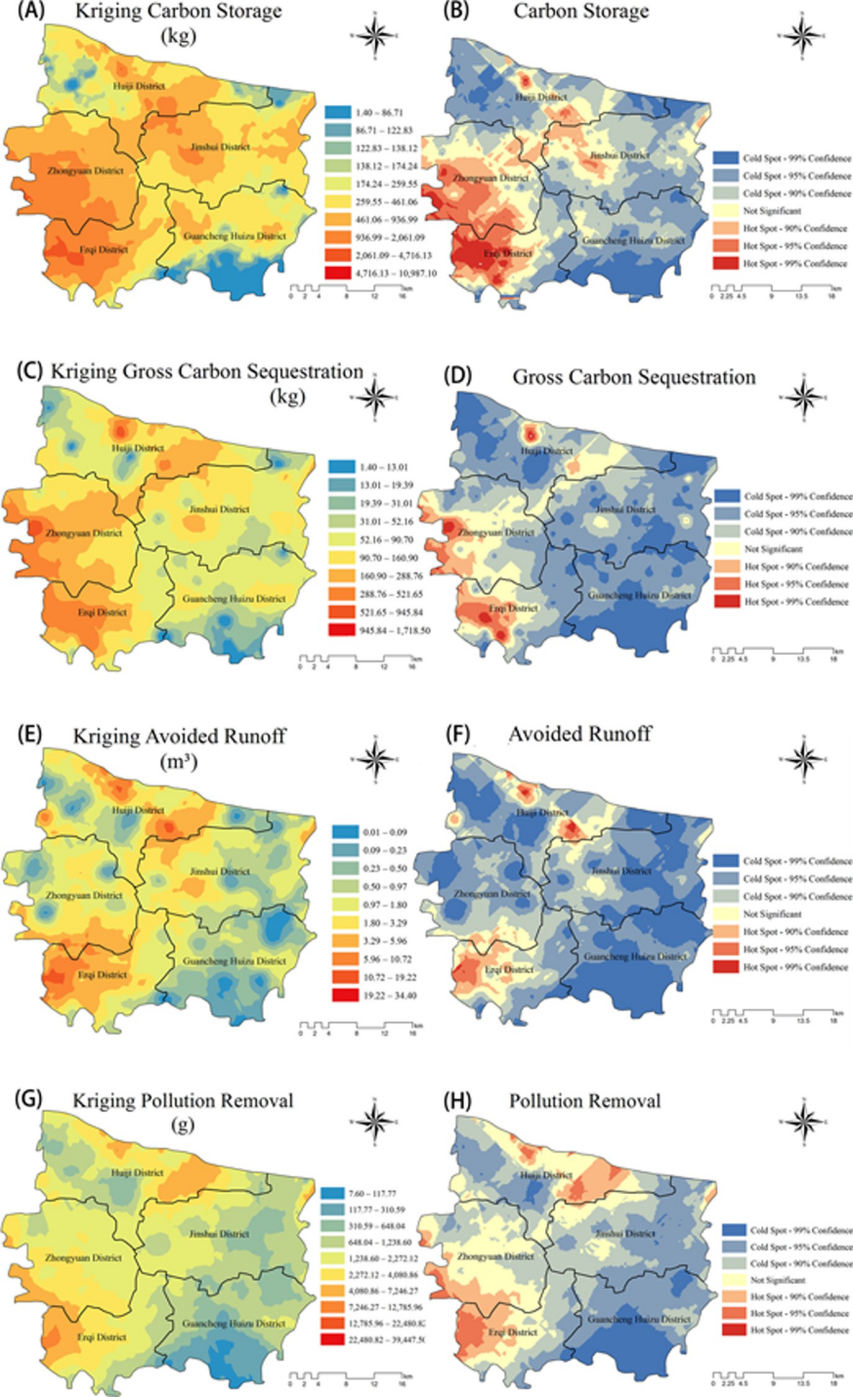
(A) Kriging interpolation of total carbon storage. (B) Analysis of hot spots of total carbon storage. (C) Kriging interpolation of annual carbon storage. (D) Analysis of hot spots of annual carbon storage. (E) Kriging interpolation of annual surface runoff avoidance. (F) Analysis of hot spots of annual surface runoff avoidance. (G) Kriging interpolation of annual pollution removal. (H) Analysis of hot spots of annual pollution removal (mnr.gov.cn).

The spatial distribution of each ecosystem service was significantly heterogeneous. In general, all ecosystem services showed the highest values in the southwestern part of the study area and the lowest values in the southeastern part. Scattered fragmented high value areas were also found in the central northern part of the study area near the Yellow River. Statistically significant high value areas were primarily located in Erqi District, and individual items such as total carbon sequestration were also partially represented in Zhongyuan District. The statistically significant low value areas were mainly located in Guancheng Huizu District and Huiji District, with Guancheng Huizu District having a larger area (Fig 6).

The spatial distributions of total carbon stock and annual carbon sequestration values were similar, both showing a wedge-shaped high value area extending from the southwest to the northwest of the city. The low values were all concentrated in the southeast and scattered in the north and east of the city (Fig 6A and 6C). Both had statistically significant high value areas concentrated in Erqi and Zhongyuan districts, with Erqi being the most numerous (Fig 6B and 6D). The difference between the two was that the total area of the high value area of the annual carbon sequestration value was significantly smaller, and correspondingly the area of the low value area was larger; the high value area was larger than the total carbon sequestration only in Huiji District, a result that may be related to the age of the trees. In addition, the total carbon storage also had a small distribution of high values in Jinshui District.

The high value areas of annual avoided surface runoff values and annual pollution removal were similarly clustered in the northern and southwestern parts of the city, and the low value areas were mostly distributed in the southeast (Fig 6E and 6G). Unlike the carbon sequestration function, the annual avoided surface runoff values were point-like and distributed in a fragmented state across the study area, while the annual pollution removal was not significant for either the highest or lowest values, with the median value occupying the widest area across the region, suggesting that there is little variation in pollution removal across the region. The statistically significant high values of annual avoided surface runoff values were concentrated in Erqi and Huiji districts, where the high values in Huiji district were more apparent than those in other ecosystem service items, while the area of high values in Erqi district was significantly smaller than others, and no statistically significant high values were found in Zhongyuan district, where all other ecosystem service items had high values (Fig 6F). The statistically significant high value area for the amount of decontamination was concentrated in the Erqi and Huiji districts and had a small distribution in Zhongyuan district, while the highest value area was the largest in Jinshui district (Fig 6H).

#### Results of integrated ecosystem service assessment

**Fig 7:**
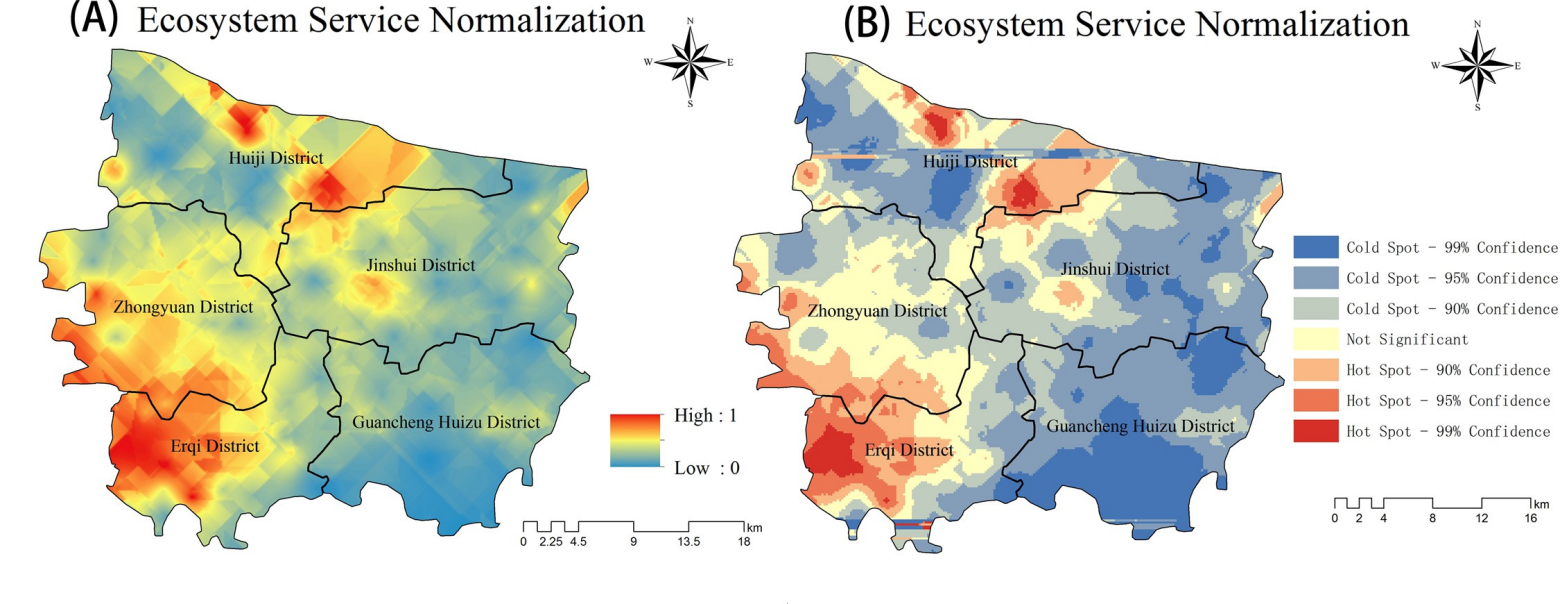
(A) Normalized map of integrated ecosystem service. (B) hot spot analysis map of integrated ecosystem service (mnr.gov.cn).

The assessment of integrated ecosystem services is to regard the three types of ecosystem services of annual carbon sequestration, annual avoided surface runoff, and annual pollution removal as having the same weight [58], and the results of integrated ecosystem service assessment were obtained by normalizing and superimposing the above three ecosystem services [78]. Integrated ecosystem services had significant spatial variability, with the major high-value aggregation area in the southwest of the city and two smaller high-value aggregation areas in the north near the Yellow River. The southeast was a low-value aggregation area with a scattered distribution in the northern and eastern parts of the city (Fig 7A). The high and low values formed an “X” shaped cross distribution in the study area. The statistically significant high value areas were located in Erqi and Huiji districts and had a small distribution in Zhongyuan district, with the largest area in Erqi district; the statistically significant low value areas were located in Guancheng Huizu district (Fig 7B), consistent with the spatial distribution results of each ecosystem service mentioned above.

### Factors influencing the spatial distribution of ecosystem services

The GeoDetector factor detector showed that all factors were significantly associated with the spatial distribution of ecosystem services (Table 2). The effect intensity (i.e., q-value) of each factor was ranked in descending order: primary industry GDP = secondary industry GDP = tertiary industry GDP > total population > land area share of forest, garden, and grassland > built-up area. The q-values of primary, secondary, and tertiary GDP were consistent and had the most significant impact. The p-value corresponding to the q-value of each indicator represents the significance of this indicator; a value less than 0.1 means significant, and the p-values of all six indicators in this study were 0, indicating that the selected indicators were all very significant.

**Table 2:** Impact indicators and results of GeoDetector on spatial distribution of ecosystem services.

Influencing Factors	q value	p value
Total Population (TP)	0.360270197	0.000
Primary Industry GDP (PIG)	0.36101386	0.000
Secondary Industry GDP (SIG)	0.36101386	0.000
Tertiary Sector GDP (TSG)	0.36101386	0.000
Built-up Area (BA)	0.110182208	0.000
Percentage of Land in Forests, Gardens and Grasslands (PFGG)	0.319068129	0.000

The GeoDetector interaction detector results showed a non-linear enhancement of the interactions between the factors (Table 3). Although the effect of these individual factors on the spatial distribution of ecosystem services was not significant, the effect was enhanced when two factors interacted; for example, the intensity of the effect increased significantly when total population and primary, secondary, and tertiary GDP interacted with other factors, the reason being that the interaction of any two variables on the spreading degree is greater than the effect of the first variable alone [71]. This also indicates that the spatial distribution of ecosystem services in urban areas of Zhengzhou is complex and is influenced by a combination of factors.

**Table 3:** Effects of GeoDetector interaction detectors on spatial distribution of ecosystem services and research results.

	TP	PIG	SIG	TSG	BA	PFGG
TP	0.360270197					
PIG	0.361037593	0.36101386				
SIG	0.361037593	0.361037593	0.36101386			
TSG	0.361037593	0.361037593	0.361037593	0.36101386		
BA	0.361037593	0.361037593	0.361037593	0.361037593	0.110182208	
PFGG	0.361037593	0.361037593	0.361037593	0.361037593	0.361037593	0.319068129

TP: Total Population; PIG: Primary Industry GDP; SIG: Secondary Industry GDP; TSG: Tertiary Sector GDP; BA: Built-up Area; PFGG: Percentage of Land in Forests, Gardens and Grasslands.

## Discussion

### i-Tree Eco

There are many methods used to assess ecosystem services. However, the model simulation method is not only a convenient tool for urban decision makers and managers but also is an important development direction for future urban ecosystem service evaluation [79].

Compared to other models, the i-Tree Eco model has several advantages in assessing urban ecosystem services: (1) the assessment of ecological benefits is made not only for tall trees but also for shrubs and grasses; (2) based on field surveys, the ecological benefits can be assessed over a large study area by the sampling method, and the results obtained are more accurate; (3) graphs and reports can be automatically generated for the study results [80]. However, this model has unavoidable limitations for international projects: (1) international users need to adjust field parameters before calculating the results, and this can lead to errors in the results. (2) The processing time for international projects is 2–6 months from the uploading of field data, so sufficient time needs to be allowed for the results to be generated.

The i-Tree Eco model uses standardized field data from randomly located plots as well as local hourly air pollution and meteorological data to quantify urban forest structure and assess its many impacts [77]. The size of the research effort will lead to different errors. In general, a stratified random sample of 200 samples will produce a standard error of about 10% in the overall urban estimates, and this decreases as the sample size increases [81]. In this study, 240 sample sites were surveyed in combination with available resources and coordinated time and manpower support, of which 118 sample sites were treeless. Although the error between the surveyed sample sites and the actual tree canopy cover in the study area was not significant, the limited nature of the research data was indeed the main reason for the error. Ma used sampling in the canopy cover area and estimated ES as the total area with canopy cover in the study area, an approach that may be able to reduce error [45].

Since fewer relevant studies have been conducted in the same region, our results are difficult to compare cross-sectionally. There are no fully locally appropriate parameters when using the i-Tree Eco model, but the software is able to account for point benefits, which facilitates spatial analysis, and thus the model is recommended more for spatial analysis rather than for specific quantitative presentation when used in international projects. In addition, we need further research on the error of i-Tree Eco estimation for international projects.

### Kriging

Geostatistics consists of two main components: the variance function and its parameters for analyzing spatial variation and structure, and Kriging interpolation for local spatial estimation. Using Kriging it should be noted that 1) the accuracy is affected by the sample point data; the more sampling points, the higher the accuracy [82]; 2) The ecological aspect of the study content itself needs to be determined before use, i.e., the spatial correlation.

Spatial variability in the type and intensity of ecosystem services is determined by the diversity of ecosystems themselves and the diversity of environmental conditions, both at macro- and micro-spatial scales [83]. Therefore, the theory of geostatistics and the corresponding methods can be applied whenever the structural and stochastic nature of spatially distributed data, spatial correlations and dependencies, or spatial patterns and variances are to be studied, when optimal unbiased interpolation estimates of these data are to be made, or when the discrete and fluctuating nature of these data are to be modeled [84]. This statement has yet to be further substantiated. The application of attempts to apply geostatistics for spatial analysis in cities is mostly seen in studies on soils [85–89]. Given the direct influence of soil on vegetation, Li et al. [44] applied geostatistical analysis in an innovative attempt to explore the distribution patterns of urban plant diversity in built-up areas of Beijing. Ma et al. [45] provided a new perspective to explore the distribution patterns of carbon storage and sequestration in urban forest vegetation using scattered data investigated in built-up areas of Beijing as an example. Based on the above study, this paper further investigated the spatial differences of urban forest ecosystem services in the Zhengzhou urban area using this method and compared the spatial correlations and errors when interpolating the four spatial values of carbon storage, carbon sequestration, surface runoff avoidance, and pollution removal. The spatial correlations of carbon storage and carbon sequestration were strong, and the spatial correlations of surface runoff avoidance and pollution removal were moderate. The MEs for the spatial interpolation of carbon storage were 0.1230, and the RMSEs were 1.0373; the MEs for the spatial interpolation of surface runoff avoidance were 0.0103, and the RMSEs were 0.2223. There were large differences in spatial correlations among different macroecological data; these have a significant impact on the accuracy of Kriging simulations, indicating that not all data could be applied to the theory and methods of geostatistics.

The use of geostatistics for spatial analysis of urban forests is not yet able to give accurate information in terms of actual quantification, but it does have the advantage of greater accuracy in the study of spatial heterogeneity and its graphical methods than previous analysis based on regional data. This provides a basis for studying spatial differences in ecosystem service flows, ecosystem supply and demand balance, and also provides a reliable basis for future urban forest planning. In addition, it can accurately show the spatial distribution pattern of ecosystem services in a larger scale, an attribute that is important for the factors affecting the spatial distribution of ecosystem service [44].

### Spatial distribution of urban forest ecosystem services and influencing factors

At present, studies on the assessment of ecosystem services in China have largely focused on mega-cities, resource-based cities, and ecologically fragile areas such as the northwest region [90]. There are few studies concerned with regional characteristics in the central region. Some researchers have studied the assessment of ecosystem services in Zhengzhou city, and the results showed that its forest ecosystem provides the greatest value of ecological services and has the strongest impact on the overall ecosystem service value [90–92]. Others have assessed the forest ecosystem services in Zhengzhou city, and the results showed that the value of forest ecosystem services is primarily related to forest area and forest structure [92–94]. Guo estimated the ecological service function of park green space in Zhengzhou city according to the theory and methods of urban forest ecosystem service assessment, and the result was also that the larger the unit area of park green space, the higher the value of its ecosystem service and the greater the ecological benefit [95]. Thus, studies on the ecosystem services of Zhengzhou City have shown the importance of urban forests. At the same time, the special locational characteristics of Zhengzhou also determine its importance. Henan Province formulated the Development Plan Outline of the Central Plains City Cluster (2006–2020) in 2005, and Zhengzhou, as the core of Zhengzhou Metropolitan Area and a typical city along the lower reaches of the Yellow River, has played an active leading role in the regional development of the Central Plains City Cluster. The results and analysis of this study may have some influence on the urban planning and development of the Central Plains City Cluster in the future.

**Fig 8:**
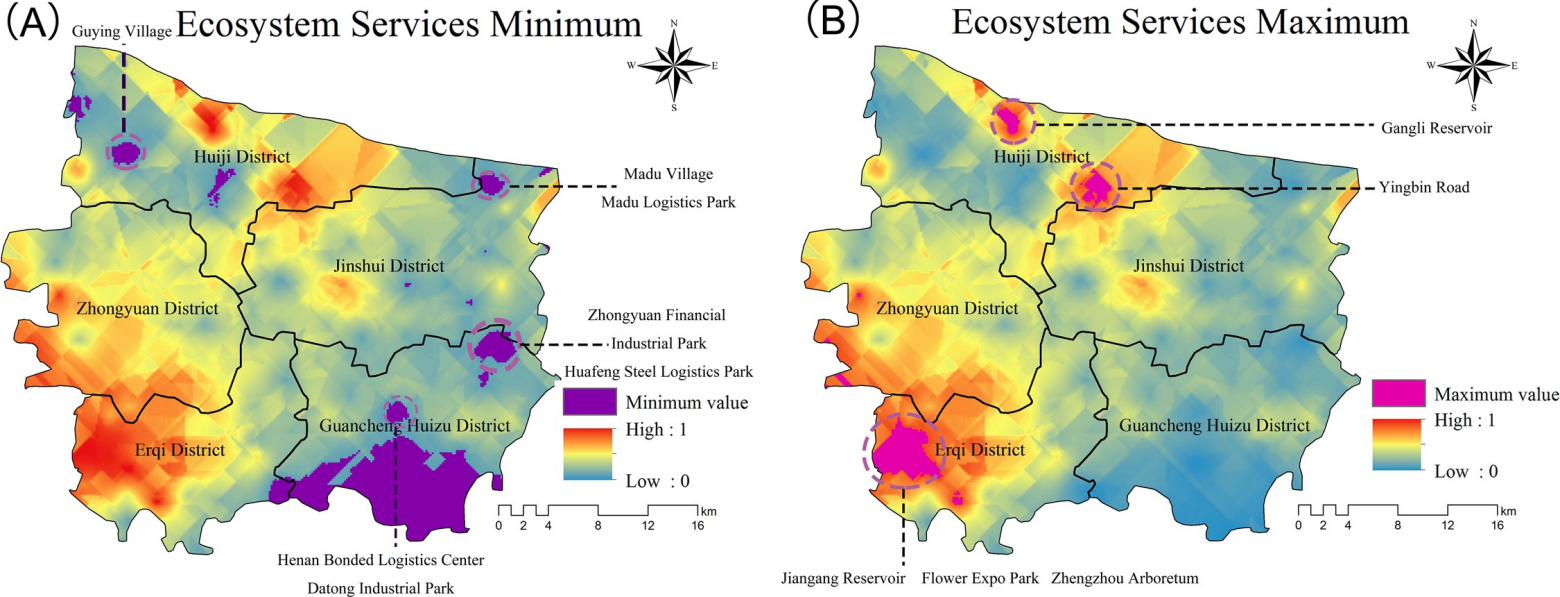
(A) Spatial distribution of the lowest value of urban forest ecosystem service. (B) spatial distribution of the highest value of urban forest ecosystem service (mnr.gov.cn).

By analyzing the influencing factors of urban forest ecosystem services in Zhengzhou, we found that the causes of typical high and low values of urban forest ecosystem services vary. From the typical low value plots, two factors representing urbanization, namely GDP and total population, are significantly and negatively correlated with urban forest ecosystem services. As shown in Fig 8A, the lowest values of urban forest ecosystem services were largely distributed in Jinshui District and Guancheng Huizu District, and most of these were located in industrial parks. This conclusion is consistent with Li et al. [96]: the value of ecosystem services in municipal and county administrative center sites, economic development zones and industrial parks is more impaired. This also confirms that the urban area of Zhengzhou City has focused on the Jinshui District and Guancheng Hui District in recent years. The most important feature of urbanization is the concentration of population, industries, and properties in cities. An increase in the urban population, changes in land use properties, an increase in the number of buildings, an increase in road pavement and impervious area, and an increase in the GDP and population are inevitable factors in the development of urbanization [97]. Measures are needed to ameliorate the degradation of ecosystem services it causes, and regional human well-being and ecological security are threatened [98]. As part of urban development planning, urban forests play a leading role in improving the urban environment and regulating the ecological balance of cities and are an important indicator of urban ecological civilization [95]. These urban green spaces—mainly urban forests—are both ecological resources that are crucial to improving people’s urban quality of life and solving socio-ecological problems, and are central to urban areas supporting biodiversity conservation and ecosystem service [99]. The vegetation growth in the urban area of Zhengzhou City showed a certain degree of positive change between 2006 and 2020, which shows that the urban forest construction in Zhengzhou City has shown a good development trend. In addition, the urban heat island in the study area also showed a trend of improvement, indicating that urban forest construction has a significant effect on mitigating urban heat islands [100].

In the typical high value plots, water, tree age, and diversity were important factors affecting the high values. According to the planning of the greening project in Zhengzhou City, it is known that the planning will focus on promoting the construction of green areas in Huiji District and Erqi District. As shown in Fig 8B, the maximum values of urban forest ecosystem services are indeed spatially distributed in the Huiji and Erqi districts, where the two high value areas are Jiangang Reservoir and Gangli Reservoir, respectively, indicating that watershed locations are beneficial for tree growth, which also makes the ecosystem service values higher in the watershed locations. This conclusion is in agreement with many research results: the shape of the high and very high ecosystem service value areas is consistent with the distribution of watersheds, and the ecosystem service value is proportional to the area of watersheds; the change in the area of woodlands has a large impact on the change in the value of ecosystem services in the study area with an amplifying effect [101, 102]. From the assessment results, another area with the highest value in Huiji District was the neighborhood near Yingbin Road, where the tree canopy coverage is extremely high, and the canopy width of individual trees is greater. The total carbon stock in this area had the highest value, but the annual carbon sequestration value was not outstanding; this may also be due to the high number of older trees. The situation is similar in the Central Plains, where the carbon sequestration function was quite high in some parts of the area, and the reduction of surface runoff was not reflected by high values. The planning of urban forests should not only consider the planting area but also include a comprehensive assessment of tree species, age, and other factors. Different tree species have different functions of ecosystem services, and urban forest planning should consider species diversity in addition to choosing high ecosystem service species as much as possible. Species diversity strategies are a key component of urban forest management [103], and maintaining high levels of biodiversity is important for the resilience of urban systems [104]. Tree age is another important factor affecting ecosystem services. The carbon sequestration and oxygen release capacity of tree species with short growth cycles are higher than those of older trees, and the uptake and release of carbon of older trees are balanced because their biomass has basically stopped increasing [105, 106]. When comparing the carbon storage capacity of individual trees, the carbon sequestration capacity of younger trees is usually not as high as that of older trees [107]. In urban greening for the purpose of pursuing ecological benefits, having more senior trees is a normal phenomenon, but the upper limit of senior trees cannot cross into the “overripe period” [108]. If there are too many old-growth trees in the “over-maturity” period, the ecological indicators of the trees will decline, resulting in a decline in ecological functions and the formation of degraded forests [109]. For urban forests, a homogeneous age structure is necessary to form a stable community and a continuous canopy cover [110, 111].

### Impact on urban planning

Fig 7 shows that the cold spot value of the built-up land area in the urban master plan is high, while the ES value is low. To improve the ecological conditions of the area and increase the ES value, the urban green space planning strategy should focus on areas such as grasslands and unbuilt land, rather than focusing on the green space area in the built land. In countries where urban forest research and practice had been carried out earlier, such as Europe and the United States, urban tree canopy cover (UTC) is the most common urban forest construction and evaluation index, and is generally divided into existing UTC and possible UTC [112]. Therefore, in future urban green space planning, the concept of potential canopy cover, i.e., land areas suitable for tree planting but that are not currently utilized for planting, can be applied, and this indicator provides a preliminary reference for industry managers and governmental decision-making departments to quantify the scale of ecological engineering from the perspective of future ecological construction. Potential tree canopy cover is of great significance for establishing ecological goals, formulating policies, and implementing concrete ecological construction in cities and their different regions; currently, it is considered a priority area for urban forest construction in countries outside of China.

### Impacts on other case studies

The research features of this paper are as follows: (1) The utilization of the i-Tree Eco model to assess and analyze the tree species characteristics of urban forests and their ES values in urban areas of Zhengzhou City. (2) A graphical analysis of the spatial heterogeneity of ES in urban forests using geostatistics, and an evaluation of the error differences in this method for different ES spatial analyses. (3) In-depth analysis of the driving mechanism of ES spatial heterogeneity with a geographic probe, as well as exploration and analysis of the typical extreme value areas of ES in urban forests in Zhengzhou City. The method of this study can be used for other cases, such as conducting multi-scale analysis. Certainly, considering the contradictory nature of urban forest and urban construction in terms of land use, urban forest ES should be assessed from a macroscopic perspective, and the regional total should be paid attention to when constructing urban forests. However, compared with previous studies conducted on large scales, fine-scale research methods make it possible to identify the problem in a more targeted way, especially in determining the contradiction between supply and demand. This approach also provides a multi-scale perspective for other future research cases, with large-scale areas for assessment and small-scale areas for planning.

## Conclusions

This study assessed urban forest ecosystem services and their spatial variation in urban areas of Zhengzhou, China, and used a Geodetector model to determine the factors affecting their spatial variation. The study showed that the spatial heterogeneity of various ecosystem services in Zhengzhou urban forest was very clear, with the highest values in the southwestern part of the study area and the lowest values in the southeastern part. In the central north of the study area near the Yellow River, fragmented areas of high values were also found. GDP and population data, which indicate the degree of urbanization, show a clear competition with urban forest construction in terms of land use; urban forestry ecosystem services are lower in urban areas with high GDP and population data. However, the urban forest ecosystem services are higher in woodland areas combined with water areas compared to pure woodlands. This will provide some referable urban construction strategies to improve both the quantity and quality of urban forest ecosystem services in the urban areas of Zhengzhou.

This paper is a further exploration of the i-Tree Eco model and Kriging interpolation based on the previous study. This is a useful attempt for spatial studies of urban forests. From the spatial correlation of the four urban forest ecosystem service indicators themselves, two indicators, carbon storage and carbon sequestration, are more suitable for the method, while surface runoff avoidance and pollution removal need to be further discussed or optimized by combining more influencing factors. Although the application of geostatistics to spatial analysis of ecology in cities has not yet achieved numerically accurate spatial quantification, improving the accuracy of spatial gradient analysis is certainly a worthwhile goal. The i-Tree Eco model and Kriging interpolation can be combined to make the evaluation of space from macroscopic to local different scales possible, effectively solving the problems of insufficient data and coarse spatial evaluation. However, it is also important to pay attention to its limitations and its high requirements for applicable data conditions, so it cannot be used blindly and requires pre-exploration of the data.

## Supporting information

S1 File (RAR)
